# Upfront Brain Treatments Followed by Lung Surgery Improves Survival for Stage IV Non-small Cell Lung Cancer Patients With Brain Metastases: A Large Cohort Analysis

**DOI:** 10.3389/fsurg.2021.649531

**Published:** 2021-10-13

**Authors:** Xiaopeng He, Shen Yin, Hongyu Liu, Rong Lu, Kemp Kernstine, David E. Gerber, Yang Xie, Donghan M. Yang

**Affiliations:** ^1^Department of Thoracic Surgery, Shandong Provincial Hospital Affiliated to Shandong First Medical University, Jinan, China; ^2^Quantitative Biomedical Research Center, Department of Population and Data Sciences, University of Texas Southwestern Medical Center, Dallas, TX, United States; ^3^Department of Statistical Science, Southern Methodist University, Dallas, TX, United States; ^4^Department of Cardiovascular and Thoracic Surgery, University of Texas Southwestern Medical Center, Dallas, TX, United States; ^5^Department of Internal Medicine, University of Texas Southwestern Medical Center, Dallas, TX, United States; ^6^Department of Population and Data Sciences, University of Texas Southwestern Medical Center, Dallas, TX, United States; ^7^Harold C. Simmons Comprehensive Cancer Center, University of Texas Southwestern Medical Center, Dallas, TX, United States; ^8^Department of Bioinformatics, University of Texas Southwestern Medical Center, Dallas, TX, United States

**Keywords:** brain metastases, National Cancer Database, treatment sequencing, surgery, non-small cell lung cancer

## Abstract

**Background:** Current treatment guidelines for stage IV non-small cell lung cancer (NSCLC) with brain metastases recommend brain treatments, including surgical resection and radiotherapy (RT), in addition to resection of the primary lung tumor. Here, we investigate the less-studied impact of treatment sequence on the overall survival.

**Methods:** The National Cancer Database was queried for NSCLC patients with brain metastases who underwent surgical resection of the primary lung tumor (*n* = 776). Kaplan-Meier survival curves with log-rank test and propensity score stratified Cox regression with Wald test were used to evaluate the associations between various treatment plans and overall survival (OS).

**Results:** Compared to patients who did not receive any brain treatment (median OS = 6.05 months), significantly better survival was observed for those who received brain surgery plus RT (median OS = 26.25 months, *p* < 0.0001) and for those who received brain RT alone (median OS = 14.49 months, *p* < 0.001). Patients who received one upfront brain treatment (surgery or RT) before lung surgery were associated with better survival than those who received lung surgery first (*p* < 0.05). The best survival outcome (median OS 27.1 months) was associated with the sequence of brain surgery plus postoperative brain RT followed by lung surgery.

**Conclusions:** This study shows the value of performing upfront brain treatments followed by primary lung tumor resection for NSCLC patients with brain metastases, especially the procedure of brain surgery plus postoperative brain RT followed by lung surgery.

## Introduction

Lung cancer is one of the most frequent sources of brain metastases (1). For non-small cell lung cancer (NSCLC), about 7–10% patients present with brain metastases at the time of diagnosis, and 20–40% of patients will develop brain metastases during their illness (2). The incidence of brain metastases is increasing with the improved availability of diagnostic imaging technology (1). Presence of brain metastases indicates stage IV NSCLC, for which the 5-year survival rate is only 5.5% (3). Patients with metastatic NSCLC are generally candidates for systemic therapy, including chemotherapy, targeted therapy, and immunotherapy (4). However, the efficacy of chemotherapy in treating brain metastases is largely limited due to the blood-brain barrier (5). On the other hand, the potential benefit of brain-local treatments, such as surgical resection and radiation therapy (RT), has been established for appropriately selected cases of stage IV NSCLC with brain metastases.

According to the National Comprehensive Cancer Network (NCCN) guidelines, stage IV NSCLC patients with limited oligometastatic disease (e.g., a single brain or adrenal metastasis) and otherwise limited-stage disease in the chest may benefit from aggressive local treatments to both the primary lung cancer and metastatic sites. Aggressive local treatment may include surgical resection and definitive RT to each site, and may be preceded or followed by chemotherapy (6). For resectable single brain metastasis, high level evidence supports category 1 recommendations for either surgical resection or stereotactic radiosurgery (SRS), followed by whole brain RT (WBRT), whereas SRS alone or following surgical resection is also a reasonable option (6–8). For single adrenal metastasis, it is only a category 2B recommendation for adrenalectomy or definitive RT (6). For solitary metastasis in organs other than the brain and adrenal glands, surgical resection to the metastatic site is still under debate.

For patients with synchronous NSCLC and brain metastases, survival benefit from resection of the primary lung tumor has been demonstrated over the past decades (9–11). Although recommended for its potential benefits, surgical intervention to the brain metastases remains a disputable option, as reflected by the frequent revisions to the guidelines (12–15). In practice, management for such a severe disease stage should be based on multidisciplinary planning. There is limited research on prognosis associated with different brain treatment options by which clinicians can plan the therapy precisely. Previously published therapeutic outcomes related to this scenario were largely based on retrospective single institution studies of highly selected cases. In the real-world clinical settings, the decision of using brain surgery in a stage IV NSCLC case is largely influenced by personal opinions and/or institutional experiences. In the present study, a total of 776 patients were carefully selected from 43,024 cases of synchronous NSCLC and brain metastases in the National Cancer Database (NCDB), generating a relatively homogenous cohort of patients who (1) had brain metastases at the diagnosis of stage IV NSCLC and (2) eventually underwent surgical resection of the primary lung tumor. Survival outcomes associated with the timing of brain-local treatments (surgery and RT) relative to lung surgery were analyzed to investigate the potentially optimal treatment plan not yet specified in the NCCN guidelines.

## Materials and Methods

This study was approved by the Institutional Review Board at University of Texas Southwestern Medical Center (STU 072016-028).

### Cohort Selection and Variable Definitions

**Figure 1:**
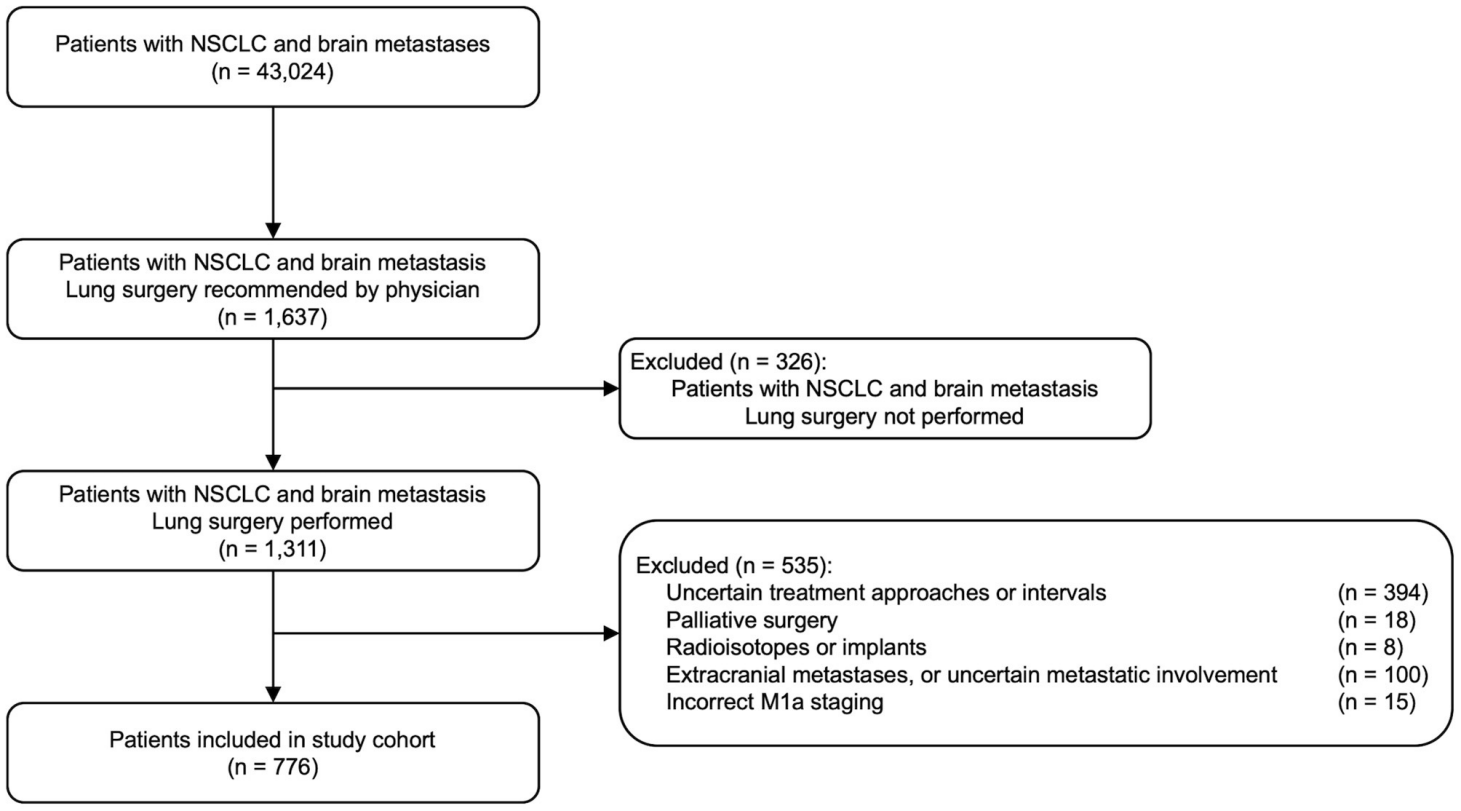
Patient cohort selection.

De-identified data for patients with stage IV NSCLC and brain metastases were obtained from the National Cancer Database (NCDB). The overall cohort selection procedure is summarized in Figure 1. Variables describing metastases, staging, the type and timing of treatments were used for patient selection and grouping (Supplementary Table 1). Specifically, histologic types of NSCLC were selected based on the 3rd edition of the International Classification of Diseases for Oncology (ICD-O-3), including squamous cell (8051–8052, 8070–8076, 8078, 8083–8084, 8090, 8094, 8120, 8123), adenocarcinoma (8015, 8050, 8140–8141, 8143-8145, 8147, 8190, 8201, 8211, 8250–8255, 8260, 8290, 8310, 8320, 8323, 8333, 8401, 8440, 8470–8471, 8480–8481, 8490, 8503, 8507, 8550, 8570–8572, 8574, 8576), large cell (8012–8014, 8021, 8034, 8082), non-small cell carcinoma (8046), and other specified carcinomas (8003–8004, 8022, 8030, 8031–8033, 8035, 8200, 8240-8241, 8243–8246, 8249, 8430, 8525, 8560, 8562, 8575) (2). The cohort was then confined to patients who had brain metastases and underwent resection of the primary lung tumor. This step effectually excluded all cases from 2004 to 2009, since the collection of brain metastases information in NCDB started in 2010. In this study, treatments of interest were limited to lung surgery, brain surgery, and brain RT. Patients were further excluded if (1) there was missing information on treatment approaches or time intervals; (2) they received only palliative surgery on either lung or brain lesions, or radioactive implants or radioisotopes; (3) they had extracranial metastases or uncertain metastatic involvement; or (4) they had inconsistent M1a staging. The metastasis staging according to the 8th edition of the American Joint Committee on Cancer (AJCC)/Union for International Cancer Control (UICC) lung cancer staging system was derived as previously described (16). Stage M1b is expected for NSCLC patients with brain metastases. Fifteen patients were excluded because they instead had M1a stage and no evidence for brain treatment, which was inconsistent with the condition of brain metastases. In addition, the variable for identifying the anatomic target of regional RT (RAD_TREAT_VOL, Supplementary Table 1) only recorded the primary site of RT if more than one region were treated. Therefore, by selecting the cases with brain RT or without any RT, patients who received lung RT were naturally excluded.

### Patient Grouping

Variables describing the type and timing of the three treatments (lung surgery, brain surgery, and brain RT) were used for patient grouping (summarized in Table 1; see also Supplementary Figure 1). In this study, lung was the primary surgery site and the distant surgery site was confined to the brain. We derived the sequence of treatments using time intervals referenced to the time of diagnosis (Supplementary Table 1). One particular group (Group 5, Table 1) included patients who received all three treatments, with lung surgery as the first treatment. In this case, the exact order of the subsequent brain treatments (surgery and RT) could not be derived based on the existing variables in NCDB. For this group, the combined brain treatments (without definite order) were denoted as Brain Surgery + RT. Details of group definitions are shown in Table 1.

**Table 1:** Patient grouping.

**Treatment combination**		**Treatment sequence**		
**Level I**	**Level II**	**Level III**	**Level IV**	**Group size**
Brain surgery	Brain surgery + RT	Upfront brain treatment	1. Brain surgery → Brain RT → Lung surgery	153
			2. Brain surgery → Lung surgery → Brain RT	60
			3. Brain RT → Brain surgery → Lung surgery	3
			4. Brain RT → Lung surgery → Brain surgery	6
		Subsequent brain treatment	5. Lung surgery → Brain surgery + RT	50
	Brain surgery		6. Brain surgery → Lung surgery	2
			7. Lung surgery → Brain surgery	2
No brain surgery	Brain RT	Upfront brain RT	8. Brain RT → Lung surgery	123
		Subsequent brain RT	9. Lung surgery → Brain RT	278
	No brain treatment		10. Lung surgery	99

*The symbol “ → ” denotes “earlier than.” The symbol “+” indicates the order of two treatments is not specified*.

### Statistical Analysis

Kaplan-Meier survival curves were generated and compared using log-rank test. A multivariate Cox regression model was used to evaluate the impact of treatments. In addition to the treatment group, we considered age at diagnosis, gender, race, facility volume, pathologic stage, type of surgical resection (sub-lobar vs. lobar) and the receipt of chemotherapy as covariates. Facility volumes were split into high and low groups by the median treatment volume of lung surgery (45 cases per year). Pathologic stage was defined based on pathologic T and N categories. Sublobar resection was identified as the surgical procedure of the primary site code ranging from 20 to 24. To further eliminate confounding, we employed propensity score stratification so that the distributions of covariates within each stratum were the same for the groups being compared. Propensity scores were estimated by logistic regression as a function of baseline age, gender and Charlson/Deyo Score. Five strata were formed based on quantiles of the estimated propensity scores. In **Figures 3, 4**, a Tarone-Ware test was used for comparing survival outcomes between treatment groups. The Tarone-Ware test has been shown to have greater power than the standard log-rank test when the proportional hazards assumption does not hold (17). All statistical tests were considered significant as *p* < 0.05. Survival analyses were implemented with R packages “survival” (version 2.43–3) and “survminer” (version 0.4.3) in RStudio (version 3.5.3).

## Results

### Characteristics of the Study Cohort

In total, 43,024 cases with NSCLC and brain metastases were identified in the NCDB, among which 1,637 cases were recommended for lung surgery by the physician. After excluding those who did not eventually receive lung surgery or did not meet other data quality criteria described above, the finalized study cohort included 776 patients (Figure 1). Table 2 summarizes the characteristics of the study cohort, separated according to whether brain surgery was performed.

**Table 2:** Demographic and clinical characteristics of the study cohort.

**Characteristic**	**Brain surgery**	**No brain surgery**
	**(*n =* 276)**	**(*n =* 500)**
**Age (year)**		
≤ 75	267 (96.74%)	444 (88.8%)
>75	9 (3.26%)	56 (11.2%)
**Sex**		
Male	144 (52.17%)	250 (50%)
Female	132 (47.83%)	250 (50%)
**Race**		
White	237 (85.87%)	425 (85%)
Black	30 (10.87%)	50 (10%)
Other	9 (3.26%)	25 (5%)
**Income (USD)**		
< $38,000	42 (15.22%)	103 (20.6%)
$38,000–$62,999	147 (53.26%)	257 (51.4%)
≥$63,000	84 (30.43%)	136 (27.2%)
Unknown	3 (1.09%)	4 (0.8%)
**Insurance type**		
None	15 (5.43%)	21 (4.2%)
Private	129 (46.74%)	195 (39%)
Public	130 (47.1%)	278 (55.6%)
Unknown	2 (0.73%)	6 (1.2%)
**Facility volume (cases/year)**		
≤ 45	151 (54.7%)	263 (52.6)
>45	125 (45.3%)	237 (47.4%)
**Charlson-Deyo score**		
0	184 (66.66%)	311 (62.2%)
1	72 (26.09%)	119 (23.8%)
2	20 (7.25%)	70 (14%)
**Chemotherapy**		
Yes	189 (68.48%)	263 (52.60%)
No	87 (31.52%)	237 (47.40%)
**Type of surgical resection**		
Sublobar resection	43 (15.58%)	174 (34.8%)
Lobectomy or larger resection	214 (77.54%)	290 (58%)
Unknown	19 (6.88%)	36 (7.2%)
**Pathologic T stage**		
T1	79 (28.62%)	116 (23.2%)
T2	112 (40.58%)	173 (34.6%)
T3	37 (13.41%)	75 (15%)
T4	12 (4.35%)	24 (4.8%)
Unknown	36 (13.04%)	112 (22.4%)
**Pathologic N stage**		
N0	154 (55.8%)	189 (37.8%)
N1	40 (14.49%)	68 (13.6%)
N2	31 (11.23%)	72 (14.4%)
N3	0 (0%)	5 (1%)
Unknown	51 (18.48%)	166 (33.2%)

### Benefit of Brain Treatments

**Figure 2:**
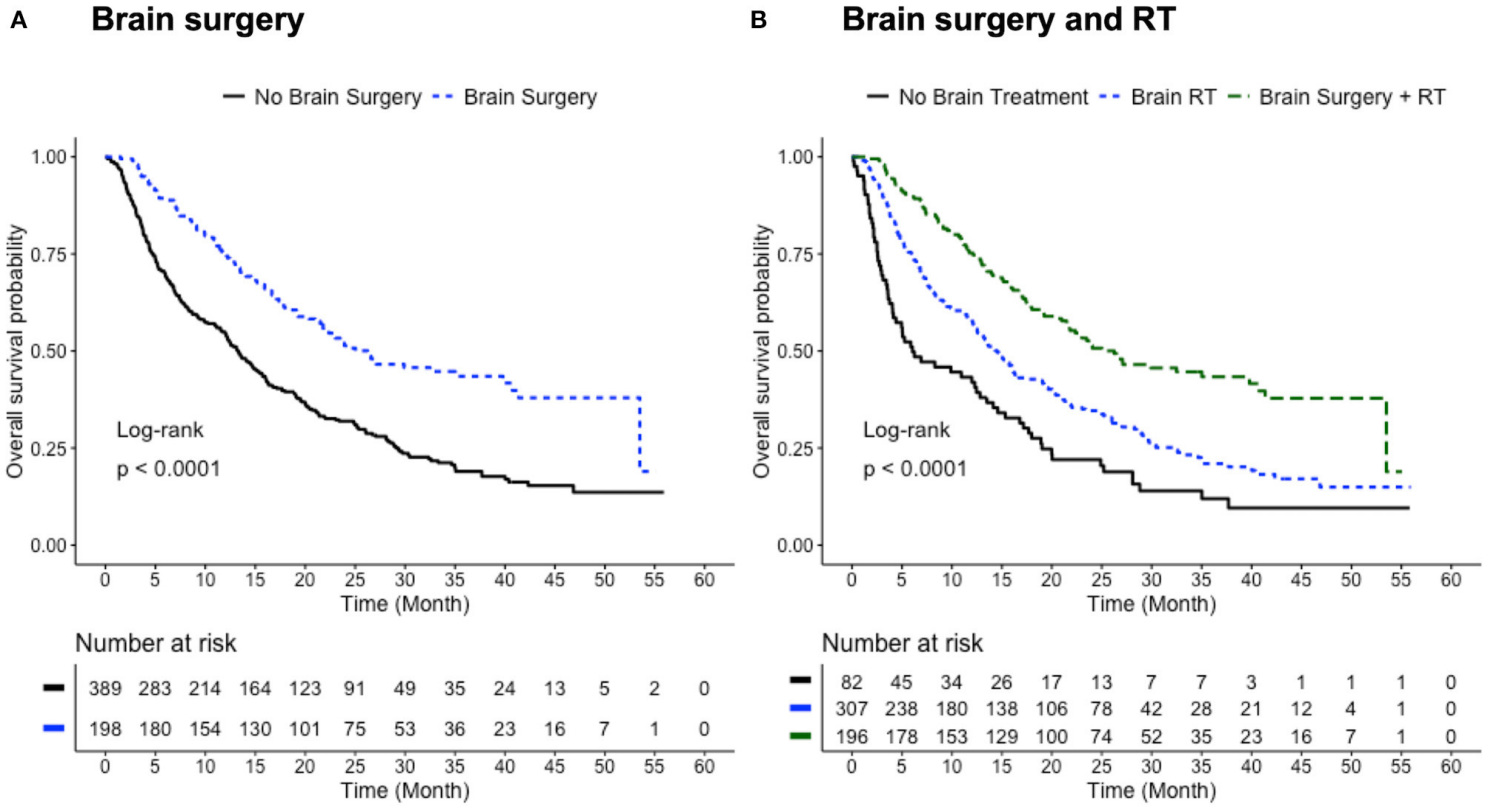
Benefit of brain treatments. **(A)** Survival curves for patients with (blue) and without (black) brain surgery. Median survival time: 26.2 months for Brain Surgery group; 13.3 months for No Brain Surgery group (*p* < 0.0001, log-rank test). **(B)** Survival curves for patients who received, in addition to lung surgery, brain surgery and RT (green), brain RT alone (blue), and no brain treatment (black). Median survival time: 26.25 months for Brain Surgery + RT group; 14.49 months for Brain RT group; 6.05 months for No Brain Treatment group. Three group comparison: log-rank test *p* < 0.0001. Pair-wise log-rank test: *p* < 0.0001 for Brain Surgery + RT vs. Brain RT; *p* < 0.0001 for Brain Surgery + RT vs. No Brain Treatment; *p* < 0.001 for Brain RT vs. No Brain Treatment. Number of patients with available survival data: *n* = 198 in Brain Surgery group; *n* = 389 in No Brain Surgery group; *n* = 196 in Brain Surgery + RT group; *n* = 307 in Brain RT group; *n* = 82 in No Brain Treatment group.

The cohort was first divided according to whether brain surgery was performed (Table 1). The patients who received brain surgery had significantly better survival than those who did not (median survival time 26.2 vs. 13.3 months, log-rank test *p* < 0.0001; Figure 2A). The survival benefit from brain surgery was also shown by propensity score stratified multivariate Cox regression (*p* < 0.0001), after accounting for the effect of age at diagnosis, gender, race, facility volume, pathologic stage, type of surgical resection and the receipt of chemotherapy.

The Level II grouping in Table 1 allows for assessing the therapeutic effects of two major brain treatment regimens: brain surgery in conjunction with RT, and brain RT alone. Patients who received only brain surgery in addition to lung surgery (*n* = 4) were not included due to the small sample size. The median survival time gradually increased with the addition of brain treatments: from 6.05 months for the No Brain Treatment group, to 14.49 months for the Brain RT group, and 26.25 months for the Brain Surgery + RT group (three-group log-rank test *p* < 0.0001; Figure 2B). All three pair-wise log-rank tests show significant differences in the overall survival within the pair. Multivariate Cox regression showed significant survival benefit for the Brain Surgery + RT group when compared with the Brain RT group (*p* < 0.001) and the No Brain Treatment group (*p* < 0.001), but no significant survival difference between the Brain RT group and No Brain Treatment group (*p* = 0.08).

### Benefit of Upfront Brain Treatments

**Figure 3:**
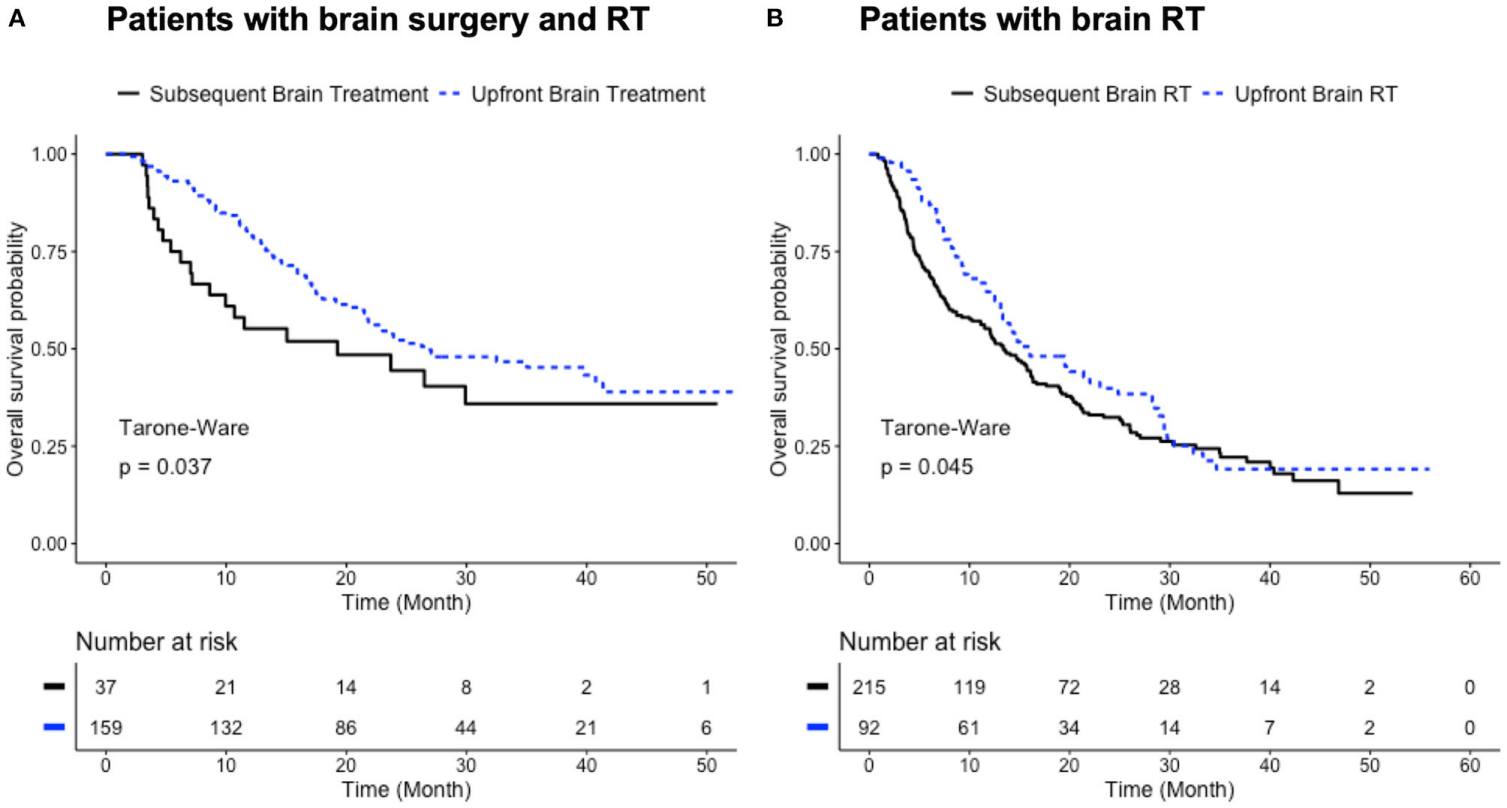
Benefit of upfront brain treatments. **(A)** Survival curves for patients who received all three local treatments with one brain treatment (surgery or RT) as the first treatment (blue) and lung surgery as the first treatment (black). Median survival time: 26.6 months for Upfront Brain Treatment group; 19.2 months for Subsequent Brain Treatment group (*p* < 0.05, Tarone-Ware test). **(B)** Survival curves for patients who received only brain RT and lung surgery (blue: upfront brain RT; black: subsequent brain RT). Median survival time: 16.0 months for Upfront Brain RT group; 13.4 months for Subsequent Brain RT group (*p* < 0.05, Tarone-Ware test). Number of patients with available survival data: *n* = 159 in Upfront Brain Treatment group; *n* = 37 in Subsequent Brain Treatment group; *n* = 92 in Upfront Brain RT group; *n* = 215 in Subsequent Brain RT.

The Level III patient grouping (Table 1) was used to study the effect of the timing of brain treatments relative to the resection of primary lung cancer. When brain RT was applied in conjunction with brain surgery, patients receiving either brain surgery or RT as the first treatment (the Upfront Brain Treatment group) had better survival than those receiving lung surgery as the first treatment (median survival time 26.6 vs. 19.2 months; Tarone-Ware test *p* < 0.05; Figure 3A). A similar pattern was also observed for patients who only received brain RT in addition to lung surgery: the Upfront Brain RT group had better survival than the Subsequent Brain RT group (median survival time 16.0 vs. 13.4 months; Tarone-Ware test *p* < 0.05; Figure 3B).

**Figure 4:**
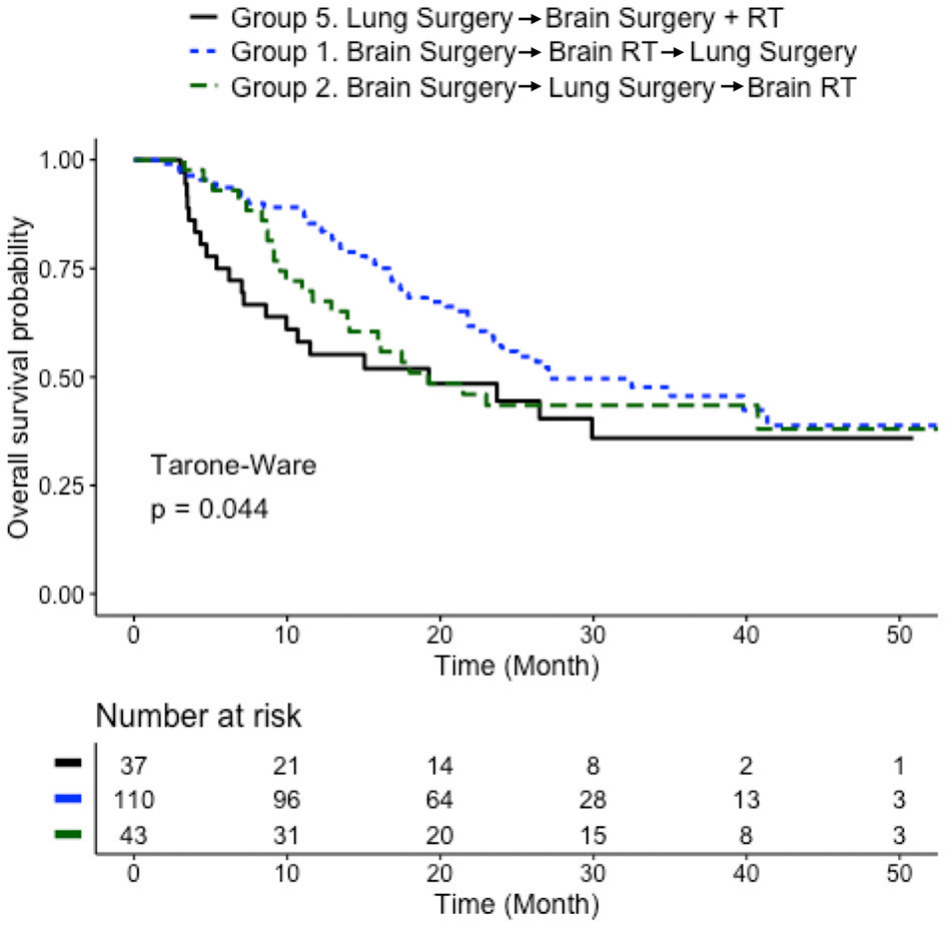
Comparison of sequences of three local treatments. Survival curves for patients who received three local treatments in different sequences (blue: brain surgery followed by brain RT and then lung surgery; green: brain surgery followed by lung surgery and then brain RT; black: lung surgery followed brain multitherapy). Median survival time: 27.1 months for Group 1; 19.2 months for Group 2; 19.2 months for Group 5. Three group comparison: Tarone-Ware test *p* < 0.05. Pair-wise Tarone-Ware test: *p* < 0.05 for Group 1 vs. Group 5; *p* = 0.14 for Group 1 vs. Group 2; *p* = 0.33 for Group 2 vs. Group 5. Number of patients with available survival data: *n* = 110 in Group 1; *n* = 43 in Group 2; *n* = 37 in Group 5.

To further study the sequence of three local treatments, three Level IV groups in Table 1 were studied: Group 1 (Brain Surgery → Brain RT → Lung Surgery), Group 2 (Brain Surgery → Lung Surgery → Brain RT), and Group 5 (Lung Surgery → Brain Surgery + RT). Patients who received brain RT as the first treatment (Groups 3 and 4; *n* = 9 in total) were excluded due to the small sample size. The group that received upfront brain surgery followed by brain RT and lung surgery (Group 1) had longer median survival time (27.1 months) than the other two groups, respectively (median survival time 19.2 months for both Groups 2 and 5). Significant difference in overall survival (Tarone-Ware test *p* < 0.05) was observed for Group 1 (Brain Surgery → Brain RT → Lung Surgery) vs. Group 5 (Lung Surgery → Brain Surgery + RT) but not in other pair-wise comparisons. When compared with Group 5 (Lung Surgery → Brain Surgery + RT), Group 2 (Brain Surgery → Lung Surgery → Brain RT) had a trend of survival benefit within the first 19 months, although not statistically significant over the whole follow-up period (Figure 4). If Groups 1 (Brain Surgery → Brain RT → Lung Surgery) and 2 (Brain Surgery → Lung Surgery → Brain RT) are combined to represent patients receiving brain surgery as the first treatment, they also had better survival compared to Group 5 (median survival time 26.6 vs. 19.2 months; Tarone-Ware test *p* < 0.05).

## Discussion

This retrospective study of NCDB data investigates the benefit of brain-local treatments and their optimal timing relative to resection of primary lung tumor for stage IV NSCLC patients with brain metastases. Management strategy for such a severe stage is underdeveloped and still lacks consensus in terms of surgical intervention to the brain. While in most circumstances the treatments at stage IV are palliative and the prognosis is very poor, survival benefit from surgical resection of primary lung tumor and brain metastasectomy has been demonstrated in patients with synchronous NSCLC and brain metastases over the past decades (9–11, 18). These findings suggest that aggressive local treatments, such as surgical resection and definitive RT, can be beneficial to appropriately selected patients, at least those with small primary tumors or without mediastinal nodal disease. According to the NCCN guidelines, patients who have a single brain metastasis and otherwise a stage I or possibly stage II lung cancer may be advised for surgical resection and definitive RT to both the primary lung and metastatic brain sites (6). However, due to limited study under such conditions, clinical practices are largely influenced by individual and/or institutional preferences.

In this study, we focused on the NSCLC patients who had eventually undergone surgical resection of the primary lung cancer, and we investigated the survival outcomes associated with different treatment plans engaging the brain metastases. In a clinical setting, individualized surgical planning is dependent on the number, size, and location of tumors, histological type, and the patient's overall health. Among the 43,024 cases with NSCLC and brain metastases in NCDB, 1,637 patients were recommended for lung surgery by the physician (Figure 1). A clear survival benefit was observed for those who eventually received lung surgery (*n* = 1,002) compared with those who did not (*n* = 262; median survival time 15.74 vs. 5.62 months; log-rank test *p* < 0.0001; Supplementary Figure 2).

In principle, resection of the primary lung lesion should be applied only to patients who have a single brain metastasis and a lung tumor that is otherwise staged at T1-2, N0-1 or T3, N0 (i.e., resectable) (6, 19). Since the NCDB data set lacks detailed information on the brain metastases (e.g., number, size, and location), in order to form a relatively homogeneous cohort regarding brain metastasis, we selected only the patients who eventually received surgical resection to the primary lung site. The fact that these patients were indeed recommended for lung surgery by the physician indicates they should have only a single brain metastasis, assuming the NCCN guidelines were followed (6). Even if considering only solitary brain metastasis, local treatment plans can be different. For a single brain metastasis, NCCN category 1 recommendations include (1) neurosurgical resection followed by WBRT, and (2) SRS followed by WBRT. SRS alone or following neurosurgery are also regarded as reasonable options, essentially giving the same priority for brain surgery plus postoperative RT and definitive RT alone (6–8). However, if the brain lesion is determined unresectable, WBRT and/or SRS can be used (7, 8). Therefore, to further minimize bias in choosing brain surgery, we separated the cohort into three major subcohorts: (1) those who received brain surgery plus RT (SRS and/or WBRT), (2) those who received brain RT (SRS and/or WBRT) without brain surgery, and (3) those who did not receive any brain treatment (Table 1, Level II). Clear survival benefits were observed for those who received brain surgery plus RT and those who received RT alone, respectively, compared to those who did not receive any brain treatments (Figure 2B). In particular, the synergistic effect of brain surgery and RT is observed as previously reported (7, 20–26). While brain surgery with postoperative WBRT has become the standard of care for solitary brain metastases (7, 20–22), similar local control of brain surgery paired with postoperative SRS has also been recognized recently (23–26). On the other hand, for limited metastases, studies of randomized trials have shown no survival benefit but increased risk of cognitive decline when adding WBRT to SRS (6, 27, 28). Interestingly, in this study only two cases involved combined SRS and WBRT and were merged into the Brain RT subcohort. The investigation of the independent benefit of brain surgery was hindered by the limited number of patients who received only brain surgery in addition to lung surgery (*n* = 4, Table 1).

As a key focus of this study, treatment sequences were investigated separately for the “Brain Surgery + RT” subcohort and the “Brain RT” subcohort (Table 1, Level II). The precise sequence of treatments was derived by analyzing the treatment time intervals (Supplementary Figure 1), a new approach in this subject. For the “Brain Surgery + RT” subcohort, where all three local treatments were performed (brain surgery, brain RT and lung surgery), an upfront brain treatment (either surgery or RT) benefited the overall survival (Figure 3A). Similarly for the “Brain RT” subcohort, where only brain RT was used in addition to lung surgery, an upfront brain RT benefited the overall survival (Figure 3B). Among the various sequences of three local treatments, brain surgery with postoperative brain RT followed by lung surgery (Group 1) appeared to be the optimal treatment plan, especially when compared with the sequence with upfront lung surgery (Figure 4). In fact, Group 1 demonstrated the longest median survival time (27.1 months) among all the groups that were tested. Performing lung surgery after the complete resection of a single brain metastasis has been advised previously (9–11). In a clinical setting, this sequence is preferred likely to observe severe neurological complications, which could render a lung surgery meaningless if it is performed before the brain surgery. Our findings in this study provide further evidence to support such clinical practices. The particular effect of an upfront brain RT in a three-treatment scenario could not be investigated due to the small sample size (*n* = 9, Table 1).

Chemotherapy is the cornerstone for the combined surgical treatment of lung cancer with synchronous brain metastases. In principle, aggressive treatment to each site may be preceded or followed by chemotherapy (6, 29). In this study, the receipt of chemotherapy was included with other covariates to avoid selection bias (i.e., patients who had better survival might simply be healthier patients, with unbalanced traits, or attributes).

## Strengths and Limitations

To our knowledge, this is the first large cohort study of the joint effect of local treatments to both the primary NSCLC and brain metastases. Due to the relative rarity of NSCLC with synchronous brain metastases and the lack of large prospective studies, clinical practices in such case are still largely influenced by the subjective opinions of clinicians and patients. This study analyzed a large cohort of NSCLC patients with brain metastases (*n* = 776) and demonstrates the particular value of performing brain treatments (surgery and/or RT) before resection of the primary lung cancer.

Adjustment for confounders, as conducted herein, may remove part but not all of the selection bias that might be present in this observational study. Since the NCDB data set lacks information about the number, size and location of brain metastases, we selected the study cohort based on the receipt of lung surgery, which should in principle apply only to patients with a single brain metastasis. To compare survival outcomes associated with different treatment sequences, we analyzed the “Brain Surgery + RT” subcohort and the “Brain RT (alone)” subcohort separately, which potentially minimizes the bias in surgical eligibility of the brain metastasis. Although apparent survival difference was observed if directly comparing these two subcohorts (Figure 2B), we avoided attributing this difference simply to the involvement of brain surgery, as patients in the Brain RT (no brain surgery) subcohort might have had brain metastasis that was not resectable. In fact, physicians are more likely to recommend brain surgery to patients with fewer, smaller, and/or more accessible brain lesions, which can exist as confounders for survival outcome. Detailed information describing the brain metastases would be desirable.

Furthermore, sample size was small for the groups receiving upfront brain RT in the “Brain Surgery + RT” subcohort (Groups 3 and 4, Table 1). This may be explained by the fact that physicians are more likely to strictly follow the NCCN guidelines and perform brain surgery (when feasible) before brain RT. In addition, sample size was small for patients who received brain surgery without RT (Groups 6 and 7, Table 1). Performing brain surgery without RT is not among the recommendations in the NCCN guidelines. RT is a relatively gentle treatment, and both postoperative WBRT and SRS are increasingly recommended to be performed in conjunction with neurosurgery (7, 20–26). This explains why most patients who had received brain surgery (*n* = 276) also had brain RT (Groups 1 through 5; *n* = 272).

## Conclusion

This study shows the benefit of upfront brain treatments for patients with synchronous NSCLC and brain metastases. For the patients who would eventually receive resection of the primary lung cancer, performing brain treatments (either neurosurgery or definitive RT) before the primary lung surgery yielded improved prognosis. The best overall survival appears to be associated with the procedure sequence of Brain Surgery → Brain RT → Lung Surgery (*n* = 153), with a median survival time of 27.1 months.

## Data Availability Statement

Publicly available datasets were analyzed in this study. This data can be found here: https://www.facs.org/Quality-Programs/Cancer/NCDB.

## Ethics Statement

The studies involving human participants were reviewed and approved by Institutional Review Board at University of Texas Southwestern Medical Center. Written informed consent for participation was not required for this study in accordance with the national legislation and the institutional requirements.

## Author Contributions

XH, DY, and YX designed the study. XH, SY, HL, and RL performed the data analysis. XH, SY, DY, and YX wrote the article. KK and DG provided critical input. All authors contributed to the article and approved the submitted version.

## Funding

This work was supported by the National Institutes of Health (Grants P50CA70907, 5P30CA1425431, R01GM115473, and 1R01CA172211); the National Cancer Institute Midcareer Investigator Award in Patient-Oriented Research (K24 CA201543-01 to DG); and the Cancer Prevention and Research Institute of Texas (RP180805). XH was a visiting scholar at the University of Texas Southwestern Medical Center and was supported by the Key Research and Development Program of Shandong Province (No. 2017GSF218096, No. 2016GSF201038, and No. 2014GSF118018).

## Conflict of Interest

The authors declare that the research was conducted in the absence of any commercial or financial relationships that could be construed as a potential conflict of interest.

## Publisher's Note

All claims expressed in this article are solely those of the authors and do not necessarily represent those of their affiliated organizations, or those of the publisher, the editors and the reviewers. Any product that may be evaluated in this article, or claim that may be made by its manufacturer, is not guaranteed or endorsed by the publisher.

## References

[1] Nayak L Lee EQ Wen PY. Epidemiology of brain metastases. Curr Oncol Rep. (2012) 14:48–54. 10.1007/s11912-011-0203-y 22012633

[2] Ali A Goffin JR Arnold A Ellis PM. Survival of patients with non-small-cell lung cancer after diagnosis of brain metastases. Curr Oncol. (2013) 20:e300–6. 10.3747/co.20.1481 23904768 PMC3728058

[3] Noone AM Howlader N Krapcho M Miller D Brest A Yu M. (editors). SEER Cancer Statistics Review, 1975–2015, National Cancer Institute. Bethesda, MD. Available online at: https://seer.cancer.gov/csr/1975_2015/, based on November 2017 SEER data submission, posted to the SEER web site, April 2018.

[4] Hanna N Johnson D Temin S Baker S Jr Brahmer J Ellis PM. Systemic therapy for stage IV non-small-cell lung cancer: American Society of Clinical Oncology clinical practice guideline update. J Clin Oncol. (2017) 35:3484–515. 10.1200/JCO.2017.74.6065 28806116

[5] Di Lorenzo R Ahluwalia MS. Targeted therapy of brain metastases: latest evidence and clinical implications. Ther Adv Med Oncol. (2017) 9:781–96. 10.1177/1758834017736252 29449898 PMC5808839

[6] Ettinger DS Wood DE Akerley W Bazhenova LA Borghaei H Camidge DR. Non-small cell lung cancer, version 1.2015. J Natl Compr Canc Netw. (2014) 12:1738–61. 10.6004/jnccn.2014.0176 25505215

[7] Nabors LB Ammirati M Bierman PJ Brem H Butowski N Chamberlain MC National Comprehensive Cancer Network. Central nervous system cancers. J Natl Compr Canc Netw. (2013) 11:1114–51. 10.6004/jnccn.2013.0132 24029126 PMC4124889

[8] Nabors LB Portnow J Ammirati M Brem H Brown P Butowski N. Central nervous system cancers, version 2.2014. Featured updates to the NCCN guidelines. J Natl Compr Canc Netw. (2014) 12:1517–23. 10.6004/jnccn.2014.0151 25361798 PMC4337873

[9] Billing PS Miller DL Allen MS Deschamps C Trastek VF Pairolero PC. Surgical treatment of primary lung cancer with synchronous brain metastases. J Thorac Cardiovasc Surg. (2001) 122:548–53. 10.1067/mtc.2001.116201 11547308

[10] Bonnette P Puyo P Gabriel C Giudicelli R Regnard JF Riquet M. Surgical management of non-small cell lung cancer with synchronous brain metastases. Chest. (2001) 119:1469–75. 10.1378/chest.119.5.1469 11348955

[11] Louie AV Rodrigues G Yaremko B Yu E Dar AR Dingle B. Management and prognosis in synchronous solitary resected brain metastasis from non-small-cell lung cancer. Clin Lung Cancer. (2009) 10:174–9. 10.3816/CLC.2009.n.024 19443337

[12] Soffietti R Cornu P Delattre JY Grant R Graus F Grisold W. EFNS Guidelines on diagnosis and treatment of brain metastases: report of an EFNS Task Force. Eur J Neurol. (2006) 13:674–81. 10.1111/j.1468-1331.2006.01506.x 16834697

[13] Kalkanis SN Kondziolka D Gaspar LE Burri SH Asher AL Cobbs CS. The role of surgical resection in the management of newly diagnosed brain metastases: a systematic review and evidence-based clinical practice guideline. J Neurooncol. (2010) 96:33–43. 10.1007/s11060-009-0061-8 19960230 PMC2808516

[14] Ettinger DS Wood DE Akerley W Bazhenova LA Borghaei H Camidge DR. Non-small cell lung cancer, version 6.2015. J Natl Compr Canc Netw. (2015) 13:515–24. 10.6004/jnccn.2015.0071 25964637

[15] Ettinger DS Aisner DL Wood DE Akerley W Bauman J Chang JY. NCCN guidelines insights: non-small cell lung cancer, version 5.2018. J Natl Compr Canc Netw. (2018). 16:807–21. 10.6004/jnccn.2018.0062 30006423

[16] Yang L Wang S Zhou Y Lai S Xiao G Gazdar A. Evaluation of the 7th and 8th editions of the AJCC/UICC TNM staging systems for lung cancer in a large North American cohort. Oncotarget. (2017) 8:66784–95. 10.18632/oncotarget.18158 28977996 PMC5620136

[17] Robert E. Tarone, Ware J. On distribution-free tests for equality of survival distributions. Biometrika. (1977) 64:156–60. 10.1093/biomet/64.1.156

[18] Paek SH Audu PB Sperling MR Cho J Andrews DW. Reevaluation of surgery for the treatment of brain metastases: review of 208 patients with single or multiple brain metastases treated at one institution with modern neurosurgical techniques. Neurosurgery. (2005) 56:1021–34. 15854250

[19] Yang CJ Gu L Shah SA Yerokun BA D'Amico TA Hartwig MG. Long-term outcomes of surgical resection for stage IV non-small-cell lung cancer: a national analysis. Lung Cancer. (2018) 115:75–83. 10.1016/j.lungcan.2017.11.021 29290266

[20] Patchell RA Tibbs PA Walsh JW Dempsey RJ Maruyama Y Kryscio RJ. A randomized trial of surgery in the treatment of single metastases to the brain. N Engl J Med. (1990) 322:494–500. 10.1056/NEJM199002223220802 2405271

[21] Vecht CJ Haaxma-Reiche H Noordijk EM Padberg GW Voormolen JH Hoekstra FH. Treatment of single brain metastasis: radiotherapy alone or combined with neurosurgery? Ann Neurol. (1993) 33:583–90. 10.1002/ana.410330605 8498838

[22] Patchell RA Tibbs PA Regine WF Dempsey RJ Mohiuddin M Kryscio RJ. Postoperative radiotherapy in the treatment of single metastases to the brain: a randomized trial. JAMA. (1998) 280:1485–9. 10.1001/jama.280.17.1485 9809728

[23] Lo SS Chang EL Sahgal A. Radiosurgery for resected brain metastases-a new standard of care? Lancet Oncol. (2017) 18:985–7. 10.1016/S1470-2045(17)30448-5 28687374

[24] Mahajan A Ahmed S McAleer MF Weinberg JS Li J Brown P. Post-operative stereotactic radiosurgery versus observation for completely resected brain metastases: a single-centre, randomised, controlled, phase 3 trial. Lancet Oncol. (2017) 18:1040–8. 10.1016/S1470-2045(17)30414-X 28687375 PMC5560102

[25] Brown PD Ballman KV Cerhan JH Anderson SK Carrero XW Whitton AC. Postoperative stereotactic radiosurgery compared with whole brain radiotherapy for resected metastatic brain disease (NCCTG N107C/CEC·3): a multicentre, randomised, controlled, phase 3 trial. Lancet Oncol. (2017) 18:1049–60. 10.1016/S1470-2045(17)30441-2 28687377 PMC5568757

[26] Kayama T Sato S Sakurada K Mizusawa J Nishikawa R Narita Y. Effects of surgery with salvage stereotactic radiosurgery versus surgery with whole-brain radiation therapy in patients with one to four brain metastases (JCOG0504): a phase III, noninferiority, randomized controlled trial. J Clin Oncol. (2018) 36:JCO2018786186. 10.1200/JCO.2018.78.6186 29924704

[27] Aoyama H Tago M Kato N Toyoda T Kenjyo M Hirota S. Neurocognitive function of patients with brain metastasis who received either whole brain radiotherapy plus stereotactic radiosurgery or radiosurgery alone. Int J Radiat Oncol Biol Phys. (2007) 68:1388–95. 10.1016/j.ijrobp.2007.03.048 17674975

[28] Chang EL Wefel JS Hess KR Allen PK Lang FF Kornguth DG. Neurocognition in patients with brain metastases treated with radiosurgery or radiosurgery plus whole-brain irradiation: a randomised controlled trial. Lancet Oncol. (2009) 10:1037–44. 10.1016/S1470-2045(09)70263-3 19801201

[29] Girard N Cottin V Tronc F Etienne-Mastroianni B Thivolet-Bejui F Honnorat J. Chemotherapy is the cornerstone of the combined surgical treatment of lung cancer with synchronous brain metastases. Lung Cancer. (2006) 53:51–8. 10.1016/j.lungcan.2006.01.014 16730853

