# Supplementary material for: Upfront Brain Treatments Followed by Lung Surgery Improves Survival for Stage IV Non-small Cell Lung Cancer Patients With Brain Metastases: A Large Cohort Analysis
Source: Front Surg. 2021 Oct 13;8:649531. doi: 10.3389/fsurg.2021.649531 (PMC8549861; doi:10.3389/fsurg.2021.649531)
Supplement: Supplementary file 1 [file Data_Sheet_1.pdf]

Supplementary Material

Supplementary Table 1.

VARIABLE	DEFINITION	PURPOSE
HISTOLOGY	Tumor histology in ICD-O-3 terms	To select all histologic types of NSCLC
CS_METS_DX_BRAIN	Distant metastatic involvement of the brain	To select patients with brain metastases
CS_METS_DX_LIVER<br>CS_METS_DX_BONE	Distant metastatic involvement of the liver<br>Distant metastatic involvement of bone	To jointly exclude patients with extracranial metastases
AJCC_V8_M_category [16]	AJCC 8th Edition Staging M category	Identify patients with M1b disease
REASON_FOR_NO_SURGERY	The reason that surgery of the primary site was not performed	To select patients for whom lung surgery was recommended but not performed
RX_SUMM_SURG_PRIM_SITE	Surgery of the primary site	To select patients receiving lung surgery
RX_SUMM_SURG_OTH_REGDIS	Surgery of the distant site	To identify the receipt of brain surgery
RAD_TREAT_VOL	Volume or target of regional radiation therapy	To identify the receipt of brain RT
DX_SURG_STARTED_DAYS	Number of days between diagnosis and the first surgery	To identify time interval before the first surgery
DX_DEFSURG_STARTED_DAYS	Number of days between diagnosis and surgery of the primary site	To identify time interval before lung surgery
DX_RAD_STARTED_DAYS	Number of days between diagnosis and RT	To identify time interval before brain RT (jointly with RAD_TREAT_VOL)
PALLIATIVE_CARE	Surgery to alleviate symptoms but no attempt to treat the primary tumor is made	To exclude patients receiving palliative surgery on either lung or brain lesions
RX_SUMM_RADIATION	Type of RT	To exclude patients receiving either radioactive implants or radioisotopes

A. Lung surgery is the first surgery

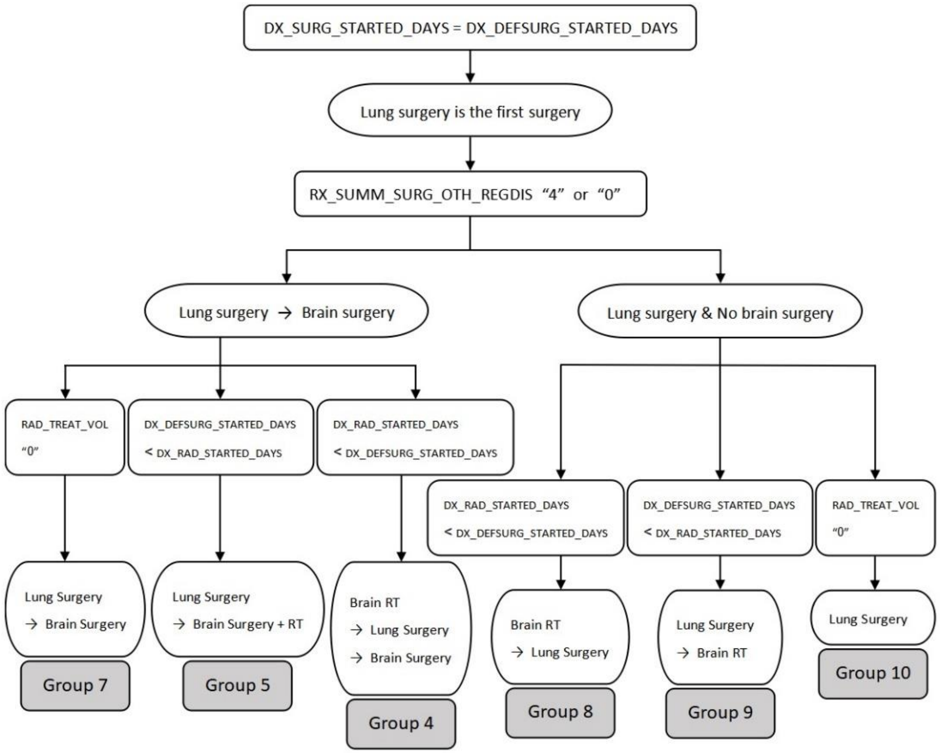

B. Brain surgery is the first surgery

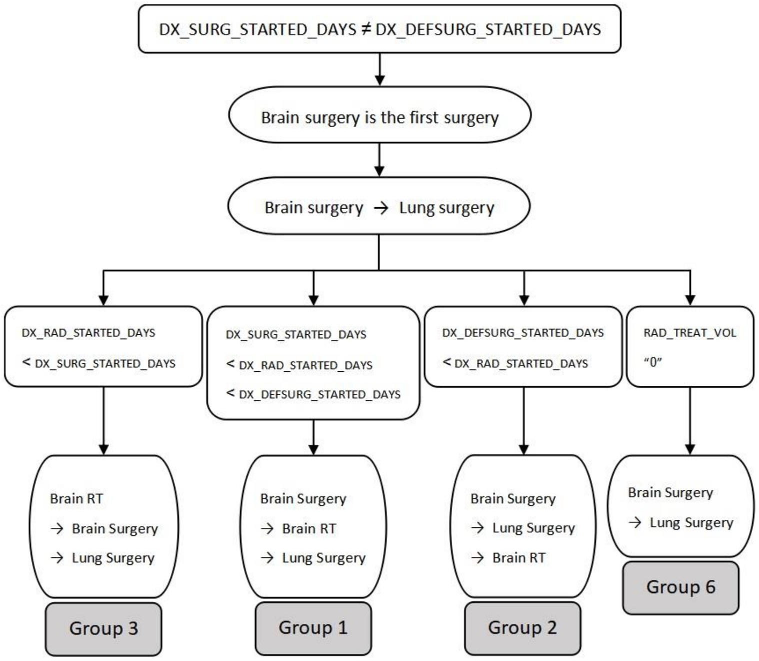

Supplementary Figure 1. Patient grouping. The symbol "⟶" denotes "earlier than". The symbol "+" indicates the order of two treatments is not specified.

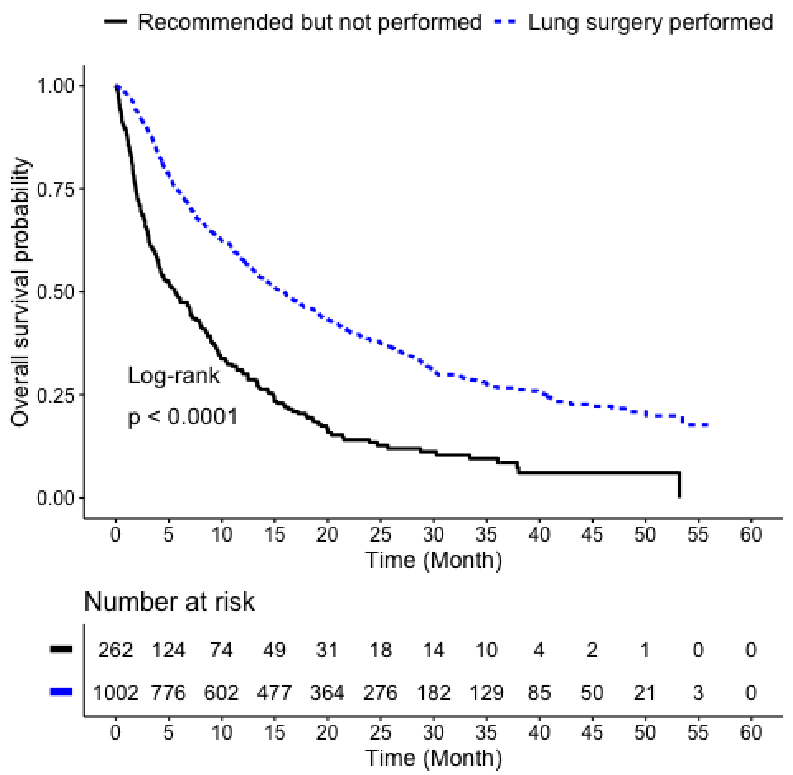

Supplementary Figure 2. Benefit of lung surgery. Survival curves for patients with (blue) and without (black) lung surgery. Median survival time: 15.74 months for patients with lung surgery performed; 5.62 months for patients with lung surgery recommended by the physician but not performed. Log-rank test $p < 0.0001$. Multivariate Cox regression with age, gender, race, facility volume and the receipt of chemotherapy as covariates p-value < 0.0001, after stratification into five levels by the estimated propensity scores. Number of patients with available survival data: n = 1002 in lung surgery performed group; n = 262 in recommended but not performed group.
